# Vestibular fold configuration during phonation in adults with and without dysphonia

**DOI:** 10.1016/S1808-8694(15)31277-5

**Published:** 2015-10-20

**Authors:** Marcos Antônio Nemetz, Paulo Augusto de Lima Pontes, Vanessa Pedrosa Vieira, Reinaldo Kazuo Yazaki

**Affiliations:** 1 Assistant Professor, Otorhinolaryngology and Head and Neck Surgery, Universidade Regional de Blumenau-FURB. Post-graduation, Program of Otorhinolaryngology and Head and Neck Surgery, Federal University Sao Paulo – UNIFESP; 2 Professor, Full Professor, Coordinator of Post-graduation, Program of Otorhinolaryngology and Head and Neck Surgery, Federal University Sao Paulo – UNIFESP; 3 Speech and Voice Therapy, Post-graduation in Internal and Therapeutic Medicine under course; Specialization in Human Communication Disorders, Federal University of Sao Paulo – UNIFESP; 4 Resident Physician in Otorhinolaryngology, Federal University Sao Paulo – UNIFESP

**Keywords:** voice, larynx, vestibular fold, laryngoscopy

## Abstract

The real participation of the vestibular folds during phonation mechanism is unknown. How vestibular folds change their configuration during phonation is still unclear. Learning about these changes in the functional mechanism of vestibular fold would be helpful for the evaluation of pathological conditions.

**Aim:**

The objective of the present study was to analyze the configuration of laryngeal vestibular folds during phonation (sustained emission of vowel/μ/) by comparing exams of individuals without vocal complaints (the normal voice group) with those with vocal complaints.

**Study design:**

Transversal simple study.

**Material and Method:**

120 images of larynges were analyzed, 60 of normal voice individuals and 60 of dysphonic subjects, with equal gender distribution. The position of the free margin of the vestibular fold was identified in relation to a straight line that brought together the anterior and posterior insertions. Regarding this position, three types of configurations were described: concave, when it was in a lateral position, convex when it was in a medial position, and linear when it overlapped.

**Results:**

Out of the 240 vestibular folds, 158 were concave, 41 convex and 31 linear. The concave form was predominant in both groups in relation to the other two forms, although the number of convex and linear forms increased in the dysphonic group. Analyzing the behavior of these forms in each gender we noticed that among women, the linear form was significantly increased in the dysphonic group, whereas among men there was significant increase in convex form.

**Conclusion:**

We concluded that there were differences in behavior of vestibular folds in the dysphonic group in relation to the normal voice group, and that the differences occurred differently in both gender groups.

## INTRODUCTION

Larynx is a muscle-cartilaginous organ with multiple functions, located in the infra-hyoid region. From a philogenetic viewpoint, the main laryngeal function is the conduction of air during breathing, followed by protecting the lower airways. In the latter, there is the sphincter function, preventing water and food input; this same function acts to prevent discharge of air from the lungs during physiological efforts, such as the act of defecting or delivering a baby, for example^1^. In the sphincter mechanism of larynx, we have the active participation of the vestibular folds and the whole vestibule. The muscles involved in closing the laryngeal lumen by vestibular folds and aryepiglottic folds is not well known, but probably is comprises the most cranial part of the thyroarytenoid muscle and also the aryepiglottic and thyroepiglottic muscles^2^. Other authors considered that the transversal fascicle of thyroarytenoid muscle forms the vestibular fold muscle. In turn, it originates from the thyroid cartilage and directs laterally in fan-like form superiorly to the vestibular fold. Mobility of vestibular fold as well as changes to pyriform sinus form would be related to elevation of vestibular fold by aryepiglottic ligament^3^, ^4^. The first description of laryngeal ventricle muscle was made by Morgagni in 1723, where two ligaments were described, one superior (vestibular fold) and another inferior one (vocal fold), in which thyroarytenoid muscle emerges and presents medium and superior fiber bundles^5^.

When the food bolus goes through the airways, there is laryngeal closure that is followed by a brief period of apnea; it lasts for few seconds, and the larynx opens immediately after for the person to breathe, whereas the bolus goes into the esophagus. If, at this moment, some food remains in the vestibule, it will be aspirated by the next inspiration movement after swallowing^6^, ^7^, ^8^, ^9^. Despite the importance of vestibular closure, it is believed that the most effective barrier against aspiration is glottic level owing to tight muscle reflex closure promoted by adductor muscles, and secondarily to the triangular format of vocal folds (seen from the front), with free margins slightly directed to cranial direction, which forms a passive valve mechanism that hinders the input of any material into the trachea^6^.

An inverse correlation may be seen when we assess vestibular folds. They have a valvular form, with free margin directed inferiorly, which imposes greater resistance to expiration flow. Thus, if it is necessary to increase subglottic and tracheal closure (cough, Valsava maneuver, for example), this disposition favors laryngeal closure^10^, ^11^. Phonation is an adapted function to the larynx, given that throughout its development it had not been designed to such purpose. There are three processes that contribute to speech production: the bellows mechanisms using air coming from the lungs, sound generation at the glottis through vocal fold vibration, resonance and sound articulation, which take place at supraglottic segment. Thus, it is not only the larynx, but the whole respiratory system and also part of swallowing movement that are important to speech production. The mechanism of speech formation at the glottic region is something very clear. It is known that aerodynamic energy generated by expiration flow is converted into acoustic energy by vocal fold vibration. To have vibration, it is necessary to have antagonistic forces that act over the vocal folds, producing opening and closure in a harmonic and successive manner. Opening force is represented by subglottic pressure. The vocal fold myoelastic force is related with laryngeal neuromuscular activity, and Bernoulli effect is one of the main sources of closure. The more mobile and elastic the vocal fold, the more intense is closure caused by the Bernoulli effect. From an ultrastructural perspective, vocal folds are organized in layers with different structural and mechanical properties, more flexible on the surface and more rigid towards the vocal muscle, which is essential for appropriate vibration movement of the vocal fold^12^, ^13^, ^14^. It is observed that there is participation of vestibular folds during the vocal production mechanism with evident changes in position and form during the process, but little or nothing is known about the meaning of this participation. In the medical literature addressing vocal production physiology, there is practically nothing about the real role performed by these structures in this complex mechanism and how these active movements change their form and contour, giving total attention to the importance of neuromuscular control and viscoelastic properties of vocal folds.

The vestibular fold is related with formation of the third formant. Formats are frequency ranges that characteristically have power centers^15^. Formant frequencies depend on vocal tract length and form. Length is defined as distance between the glottis and lip opening. Initial glottic sound is modified by resonance. Human resonance system depends directly on three-dimensional geometric configuration of vocal tract and its walls^16^. The movement of one of the articulators normally affects the frequency of all formants. The frequencies of the first formant are particularly sensitive to changes in mandible lowering. The increase of mandible lowering tends to increase frequency of the first formant. The second formant is particularly sensitive to changes in tongue posture. When the tongue makes a constriction point in the anterior portion of the vocal tract, the frequency of the second formant is higher. If the tongue makes a velar constriction point, frequency of the second formant is lower. The second formant reaches its lowest value when the tongue touches palatine veli and lips are protruded, such as to produce vowel/u/. The third formant is related to resonance of the region above the vocal folds, formed by laryngeal ventricles, aryepiglottic folds and vestibular folds^17^. We know that this formant is particularly sensitive to tongue tip positions, or more specifically the size of cavity that is formed immediately after lower incisors. If this cavity is wide, the frequency of the third formant tends to be low^18^. Mean values of frequency of formants found in Brazilian Portuguese native speakers for the first, second and third formants of oral vowel/a/are respectively 956, 1634 and 2721 Hz; for oral vowel/i/they are respectively, 425, 2984 and 3668 Hz, and for oral vowel/u/, 462, 1290 and 2528 Hz, with significant standard deviation between analyzed subjects^19^.

Anatomically, vestibular folds are inserted in the highest portion of the internal angle of the thyroid cartilage following horizontally to the back, fixing on the internal aspect of the arytenoid cartilage. It has the format of two flat laminas, with two aspects and two margins. The upper aspect, tilted down, corresponds to the laryngeal supraglottic portion. The lower aspect follows the same direction and form of the internal wall of the ventricle. The external margin continues with the aryepiglottic fold and the internal or free margin follows the glottic rhyme. The vestibular fold structure is formed by the upper thyroarytenoid ligament, which is a fibroelastic lamina^20^, ^21^, ^22^, ^23^. The subepithelial layer of vestibular folds has more glands (128/cm^2^) than the glottic region (13/cm^2^), reason why it is important to lubricate the laryngeal epithelium and the viscoelastic properties. Vestibular folds during phonation mechanism apply strong tension to arytenoid cartilages and simultaneously pull upwards aryepiglottic folds, widening the ventricle and providing space to higher amplitude of vocal fold vibration^24^. Phonation disorders associated with vestibular fold structure are related in the medical literature as ventricular dysphonia or vestibular dysphonia^25^, ^26^, ^27^. This type of dysphonia may occur as a compensation owing to laryngeal anatomical or physiological affections or as an isolated hypertrophy of vestibular fold^28^. In vocal fold paralysis, depending on the position of the paralyzed vocal fold, there may be contralateral vestibular fold medialization. Midline paralyzed folds present medialization of vestibular folds in 59.5% of the cases, given that in lateral positions it takes place in 77.7% of the times. It occurs because of the compensation adjustment of the vestibular folds owing to insufficient glottic coaptation, that is, as the glottic chink increases, the vestibule attempts further to compensate by developing an additional sound source^29^, ^30^, ^31^. It is assumed that the same may happen in other conditions that affect the glottic chink. The scarcity of studies addressing vestibular folds in vocal mechanism is justified by the rarity of studies that addressed the topic. We understand that knowledge of the participation of vestibular folds in laryngeal physiology may have practical applications, because it will allow better assessment of functional impairment in different conditions, which will support the definition of strategies for appropriate treatment.

## OBJECTIVE

Check whether there is difference in the form of vestibular folds in a group of patients with vocal complaints when compared to a group of patients without vocal complaints, considering gender differences.

## MATERIAL AND METHOD

The material in the present study comprised one hundred and twenty image recordings of larynges obtained with videolaryngoscopy conducted at Instituto da Larynge - INLAR, Sao Paulo. The larynges belonged to adult subjects aged over 18 years and below 45 years distributed equally between male and female subjects, 60 of them without vocal affections, comprising the normal voice group, and 60 with vocal affections, comprising the dysphonia group. Each 10 female subjects with vocal complaints had one of the three types of affection: vocal nodule, unilateral vocal fold paralysis, and minor structural alterations (vocal fold cyst or stria sulcus). Male subjects, similarly to female ones, had granuloma, unilateral vocal fold paralysis and minor structural alterations. Vestibular folds were analyzed individually, which resulted in 120 vestibular folds in each one of the two groups. There were no specific procedures for patients in this study, because they were file images. Telelaryngoscopy was conducted under topical anesthesia with Lidocaine 10% spray, the patient was seated and had his/her mouth opened, with protruded tongue maintained by digital pressure, involved in gauze. It was conducted using telescope Machida® 70º and conventional video system with microcamera Panasonic® KS152. During assessment, the patient was instructed to breathe through the mouth, without effort and to produce sustained vowel//, at intensity and frequency close to habitual emission. Images were transferred to a computer AMD Atlon XP 1600 1.6®, with operational system Windows 2000®, and digitalized using image capturing program Adobe Premiere^*®*^ with video board Pinnacle® DC 1000. Out of these images we collected only one frame for phonation of sustained//, discarding the beginning and end of emissions.

Upon randomly selecting the images from the file, we considered the following exclusion criteria:

• Presence of vocal fold lesions except for nodules, granulomas, paralysis or minor structural alterations such as cyst or stria sulcus;
• Inability to localize insertion of vocal fold under direct view through laryngoscopy or interference through frame by frame dynamic analysis;
• Poor quality of filed image or presence of discharge that did not allow definition of limits of the different structures;
• Images in which emission occurred with the presence of nausea reflex.

**Figure 1:**
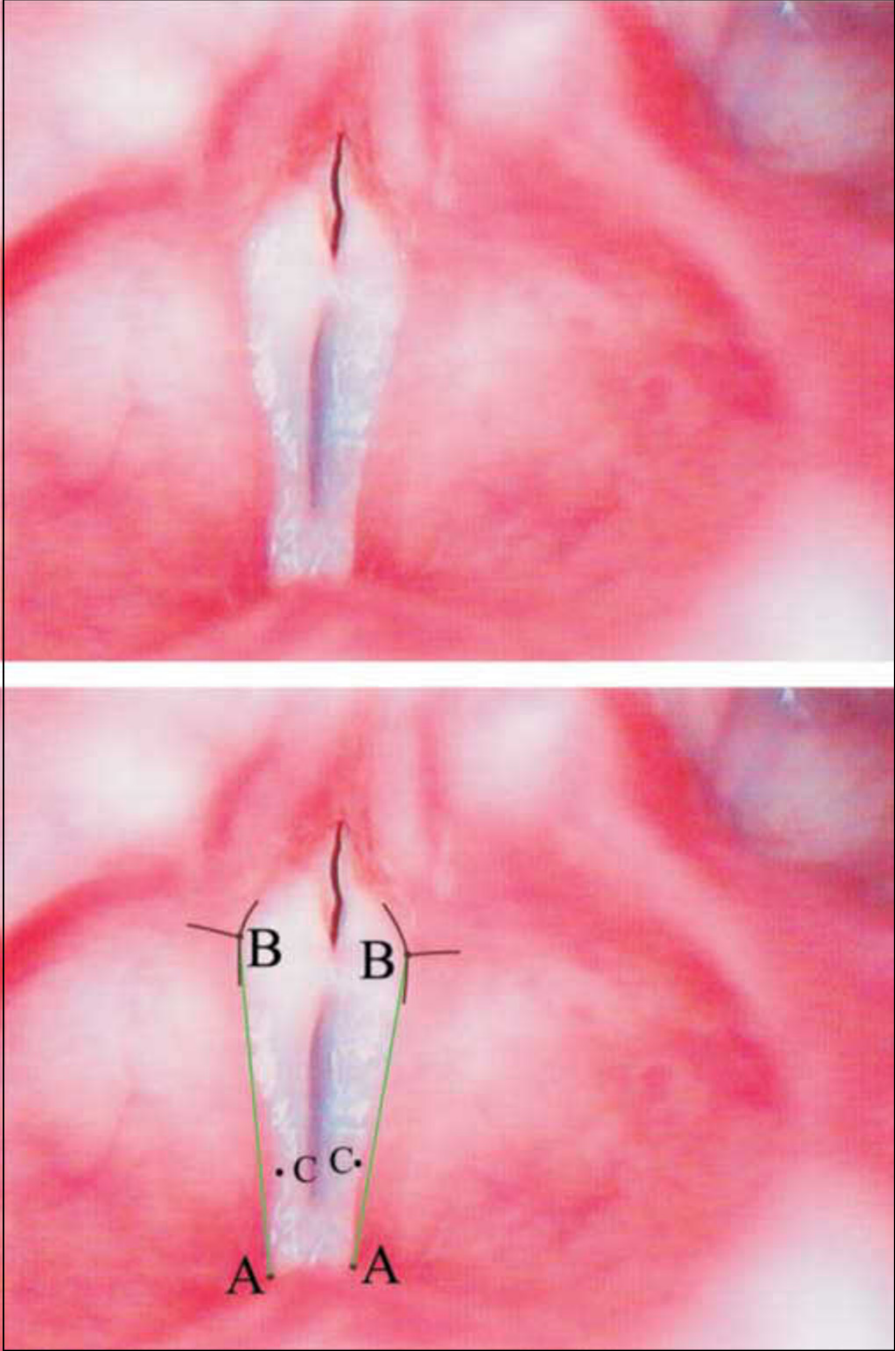
Superior – Laryngeal telelaryngoscopic image in male gender during emission of vowel/e/with major stria sulcus and Convex vestibular fold. Inferior – same image of points, definition insertion lines of points B (black) and straight reference lines AB (green).

These images were analyzed in the computer to define reference parameters. Structures were identified by differences in staining and/or their respective intensities. Three anatomical points were identified to reach definition of vocal fold configuration, to wit: anterior, posterior and displacement points (Figure 1).

**Anterior Point** (A) –corresponds to anterior insertion of the vestibular fold margin close to internal angle of thyroid cartilage (Figure 1). It is variable, and may be close to the petiole of epiglottis or far away from it. In other occasions, before it reaches the region, the margin is dichotomized and forms a triangle; in these cases, we considered the opening as the insertion point (Figure 1).

**Posterior Point** (B) –it is the posterior end of the vestibular fold free margin. It is identified by using as a reference the surface of arytenoid region, surface of vestibular folds and surface of vestibular aspect of vocal fold. The junction of these three surfaces formed three conjoining lines, whose intersection point was named posterior point B: a lateral line between the darkest aspect of arytenoid region with the clearest surface of vestibular fold, posterior-medial aspect that corresponded to the junction of the darkest surface of arytenoid region against the clearest and brightest surface of vocal fold, anterior-medial portion formed by the contrast between the vestibular fold margin and the vocal fold surface. Upon determining it, the contrast was fundamentally defined by color and not intensity.

**Figure 3:**
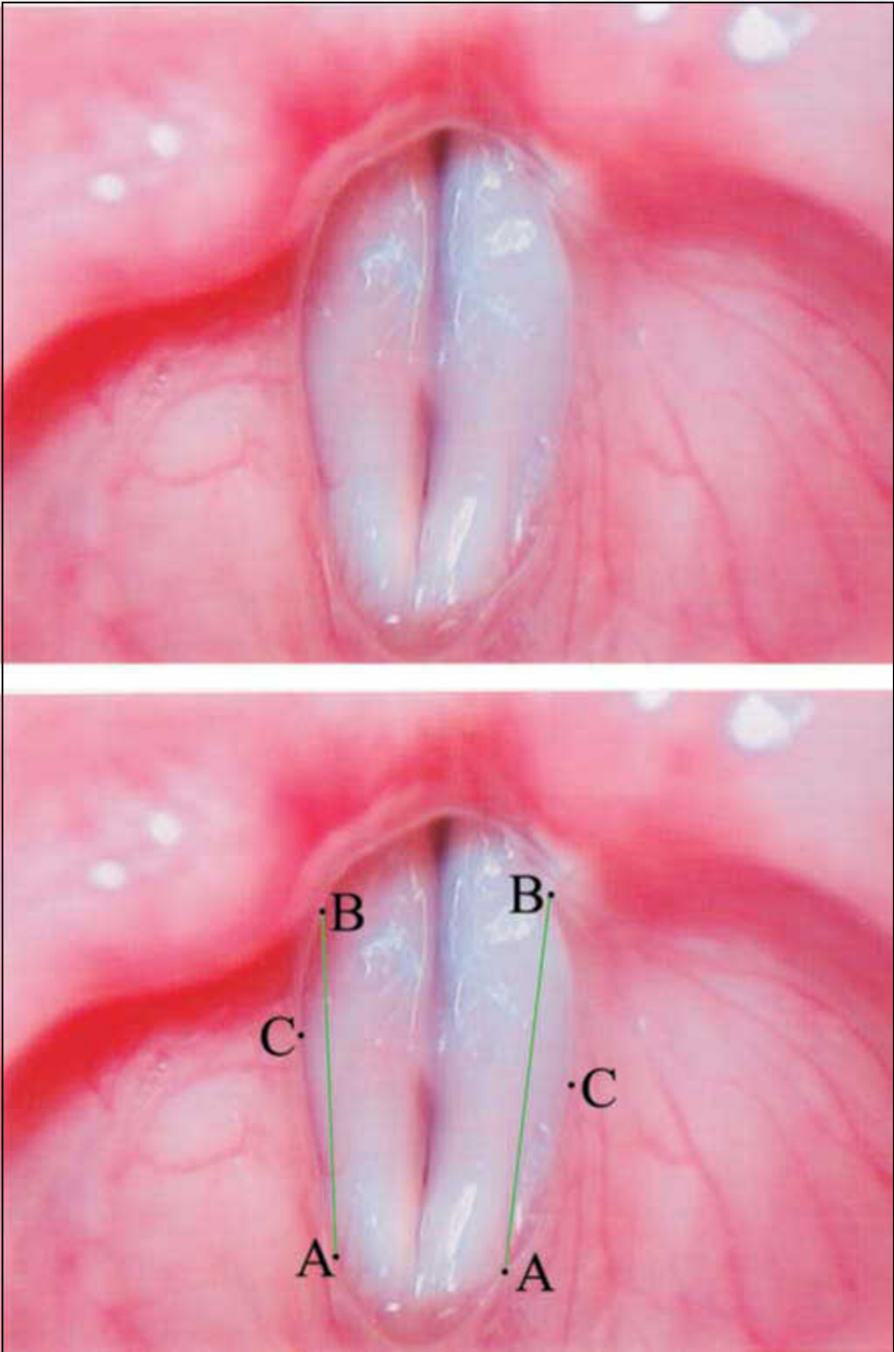
Superior – Laryngeal telelaryngoscopic image of female gender during emission of vowel/e/with minor stria sulcus and Concave vestibular fold. Inferior – same image of points and reference arrows AB.

The line that joins both points was denominated AB (Figure 3).

When these two points or one of them was not exposed by telelaryngoscopy, we observed adduction movement frame by frame and if it was possible to infer the position of the insertions, the image was considered ready for the analysis.

**Figure 2:**
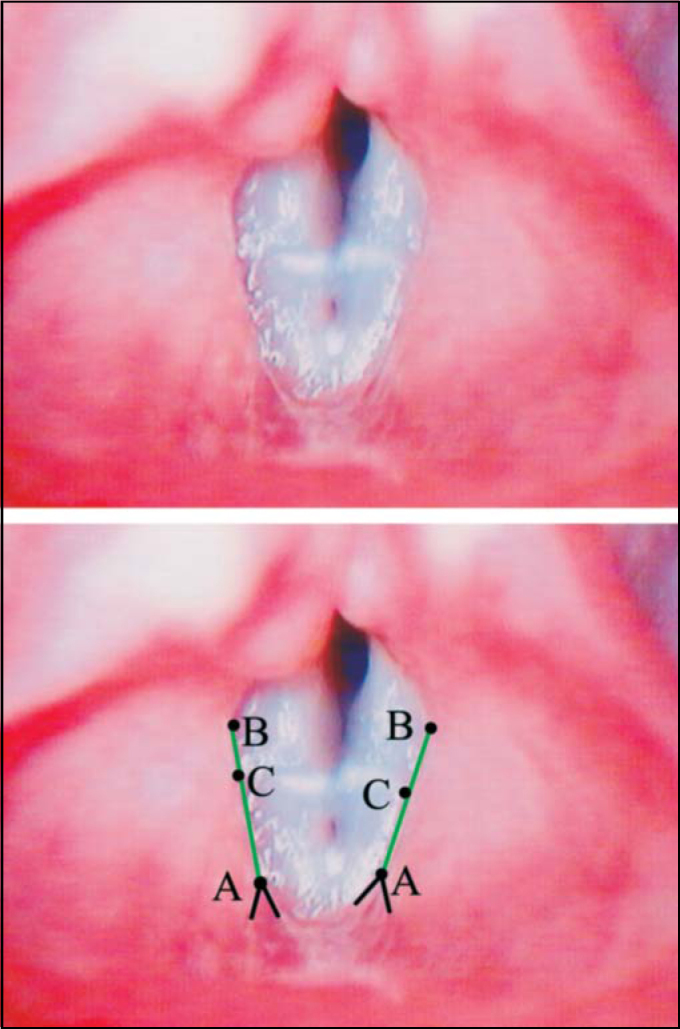
Superior – Laryngeal telelaryngoscopic image in female gender during emission of vowel/e/with vocal nodule and linear vestibular folds. Inferior – same image of points (black), definition lines of points (black) and straight reference lines (green).

**Figure 4:**
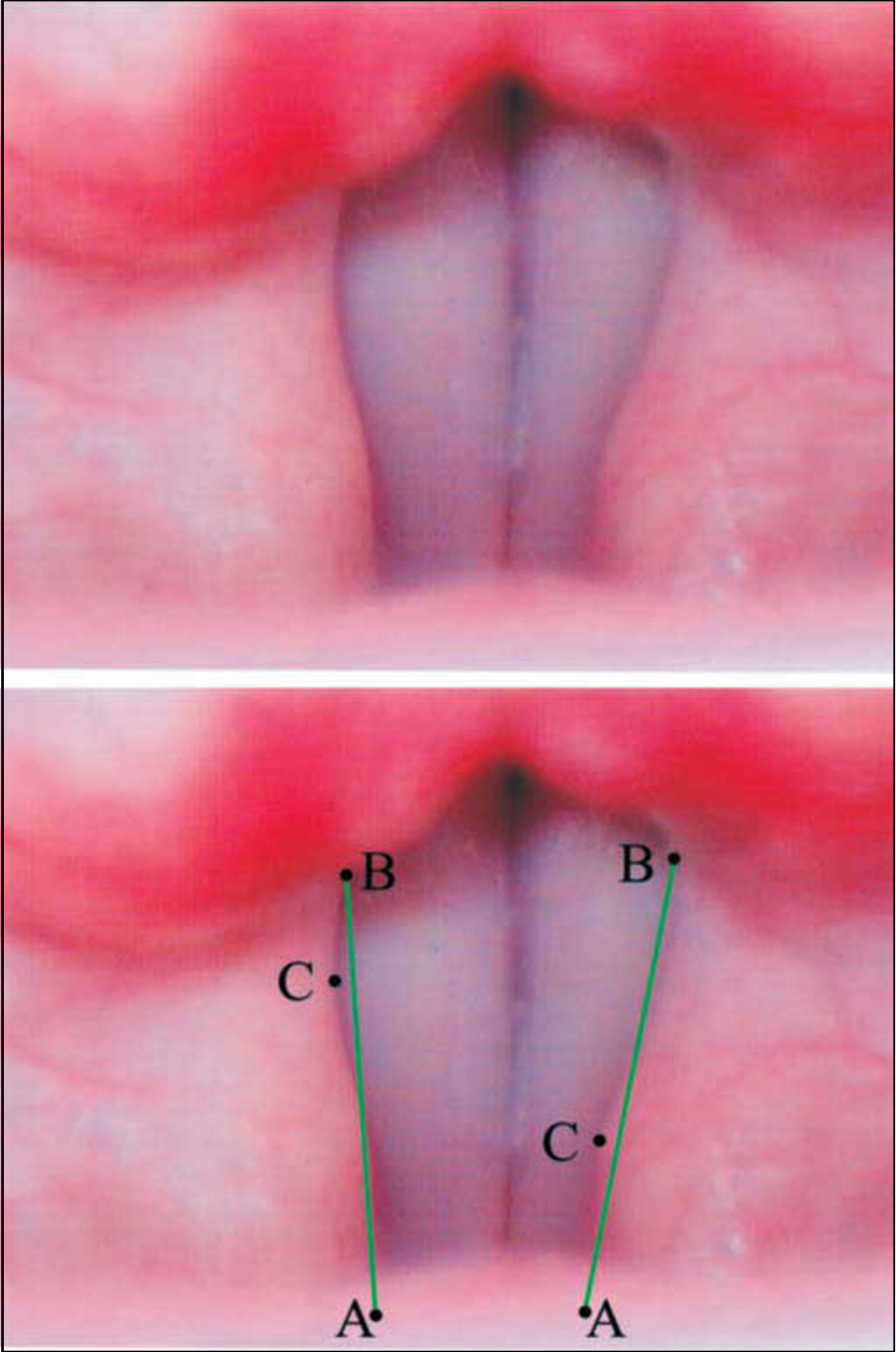
Superior – Laryngeal telelaryngoscopic image of male gender during emission of vowel/e/, normal voice, with sinusoid vestibular folds, right one Concave and left one Convex. Inferior – same image of points and reference arrows AB.

**Displacement Point** (C) is the point that corresponds to higher displacement of vestibular fold in relation to line AB (Figure 1, Figure 2, Figure 3, Figure 4).

According to the position of point C in relation to line AB, we could define three types of vestibular fold free margin:

• **Convex** form – when point C of vestibular fold free margin was medially located to AB line (Figure 1).
• **Concave** form – when point C of vestibular fold free margin was laterally located to AB line (Figure 3).
• **Linear** form – when point C was overlapped or very close to line AB (Figure 2).

When the vestibular fold free margin had lateral and medial part located in relation to line AB (sinusoidal), it was considered Concave or Convex according to the position of point C (Figure 4).

Statistical analysis: we used non-parametric analysis; if there were differences, we employed parametric tests:

• Two-sample test for proportion (t test for two samples of equivalent and different variables, and paired t-test);
• Adherence Test (chi-square test) –for frequencies of the same group. Significant results were identified with asterisks.

## RESULTS

The results are presented in tables.

## DISCUSSION

Results showed that Concave form of vestibular folds during phonation is the standard form, considering both genders and presence or not of dysphonia (Tables 1 and 3). However, comparing the results in Table 2, we noticed that in the dysphonia group the reduction in number of Concave form resulted in increase in Convex and Linear forms. Once differences in relation to dysphonia were configured, the question we asked was whether the difference really occurred similarly for both genders. Table 4 shows that in males, there was significant increase in convex form in the dysphonia group, whereas in females, this increase was noticed in the linear form. Based on the results, we may assume that when phonation is harmonious, vestibular folds behave similarly in both genders, but if there are difficulties, they behave differently concerning form, but similarly in terms of displacement, that is, from lateral to medial. The next questions is then: why do women vestibular folds move differently concerning form when compared to men with dysphonia? We do not have an answer but we may suppose that the difference lies in the laryngeal anatomical configuration that differs in both genders and is clinically translated as glottic proportion differences. We know based on previous studies^33^ that low glottic proportion, such as in female pattern larynges, has incomplete narrowing of arytenoids, which results in presence of posterior chink during phonation in women and complete approximation in men, in whom posterior chink is rare^32^. It is possible to hypothesize that this difference in anatomical configuration is also related with vestibular fold forms, depending on gender in dysphonia; phonation efforts would act to medialize vestibular folds, resulting in different conformations of vestibular folds in both genders. In men, the medialization would be favored as in the arytenoids, and therefore, the convex form would predominate in males, what corresponds to predominance of linear form in females. If this hypothesis is confirmed in future studies, simple observation of vestibular fold configuration would be a valuable semiological signal that, added to other signals, would support us in the diagnosis of presence of strain during phonation, even before the patients presented vocal quality affections. In addition to the possible semiological value of vestibular fold configuration, we could also related it with physiological aspects that involve the 3rd and 4th formants, similarly to singers' formants. There are considerable differences between voices in every day talk and those in operatic or concert singing. These differences are not only musical expression but also serve to produce greater vocal power in the presence of orchestra sounds. This increase in power is related with onset of reinforcement in the envelope of acoustic spectrum in the region 2-3KHz, coinciding with the range of 3rd and 4th formants, as if there were a fusion between both; in male singers this reinforcement is very evident and know as singer's formant. For the occurrence of fusion between 3rd and 4th formants, three simultaneous events should take place^17^, ^18^:

1. Transversal section of pharynx in the laryngeal region has to be six times higher than the area of laryngeal opening;
2. Laryngeal lowering;
3. Increase in laryngeal ventricle.

**Table 1:** Numeric distribution of vestibular folds, according to configuration of normal voice group and dysphonia group and genders.

Form	normal voice group		dysphonia group		Total
	Female	Male	Female	Male	
Concave	51*	49**	36***	32****	168
Convex	08	02	10	21	41
Linear	01	09	14	07	31
Total	60	60	60	60	60

* χ^2^ = 14.68 p = 0.00065 g.l. = 2

** χ^2^ = 8.41 p = 0.01493 g.l. = 2

*** χ^2^ = 14.08 p = 0.00088 g.l. = 2

**** χ^2^ = 19.51 p = 0.00006 g.l. = 2

**Table 3:** Numeric and perceptual distribution of Concave form of vestibular folds in normal voice and dysphonia groups in relation to genders.

Gender	normal voice group		dysphonia group		Total	
	N	%	N	%	N	%
Male	49	49%	32	47.06%	81	48.71%
Female	51	51%	36	52.94%	87	51.79%
Total	100	100%	68*	100%	168	100%

* χ^2^ = 0.06 p = 0.805 g.l. = 1

**Table 2:** Numeric and percentage distribution of vestibular folds according to configuration in relation to normal voice and dysphonia groups.

Forma	normal voice group		dysphonia group		Total	
	N	%	N	%	N	%
Concave	100	83.3	68	56.6	168	70.0
Convex	10	8.3	31*	25.8	41	17.0
Linear	10	8.3	21*	17.5	31	12.9
Total	120	100	120	100	240	100

* χ^2^ = 20.75 p < 0000.3 g.l. = 2

**Table 4:** Numeric and perceptual distribution of convex form of vestibular folds in normal voice and dysphonia groups in relation to genders.

Gender	normal voice group		dysphonia group		Total	
	N	%	N	%	N	%
Male	02	20%	21*	67.74%	23	56.10%
Female	08	80%	10	32.26%	18	43.90%
Total	10	100%	31	100%	41	100%

* χ^2^ = 5.19 p = 0.0227 g.l. = 1

Considering that the lateral wall of the ventricle is fixed and that the floor during phonation is formed by vocal folds in coaptation, it is evident that increase of ventricle can only occur by the mobilization of the vestibular folds towards medial direction, or taking on a convex form as occurs in men, satisfying item 3. On the other hand, this vestibular fold behavior will result in reduction of laryngeal lumen in the region of vestibular rhyme, what will elevate the relation between pharyngeal transversal section in the laryngeal region and laryngeal lumen area, which also satisfies the requirement of item 1 for fusion of 3rd and 4th formants. Thus, we can infer that this anatomical-functional characteristic favors formation of singer's formant in male gender.

**Table 5:** Numeric and perceptual distribution of linear form of vestibular folds in normal voice and dysphonia groups in relation to genders.

Gender	normal voice group		dysphonia group		Total	
	N	%	N	%	N	%
Male	09	90%	07	33.33%	16	51.61%
Female	01*	10%	14*	66.67%	15	48.39%
Total	10	100%	21	100%	31	100%

* χ^2^ = 8.71 p = 0.000316 g.l. = 1

## CONCLUSION

In view of the collected data, we concluded that there were changes in vestibular fold behavior in the group of patients with dysphonia compared to the group without vocal complaints. In men, modification occurred as a result of significant increase in the convex form, whereas in women it resulted from linear form, but in all situations, concave was predominant.
